# A new predictive model for the concurrent risk of diabetic retinopathy in type 2 diabetes patients and the effect of metformin on amino acids

**DOI:** 10.3389/fendo.2022.985776

**Published:** 2022-08-18

**Authors:** Zicheng Song, Weiming Luo, Bing Huang, Yunfeng Cao, Rongzhen Jiang

**Affiliations:** ^1^Department of Obstetrics and Gynecology, Shanghai Jiao Tong University Affiliated Sixth People’s Hospital, Shanghai, China; ^2^Department of Toxicology and Sanitary Chemistry, School of Public Health, Tianjin Medical University, Tianjin, China; ^3^Research Department, Dalian Innovation Center of Laboratory Medicine Mass Spectrometry Technology, Dalian, China; ^4^Research Department, Clinical Mass Spectrometry Profession Technology Innovation Center of Liaoning Province, Jinzhou, China; ^5^Research Department, Dalian Laboratory Medicine Mass Spectrometry Technology Development Innovation Team, Dalian, China; ^6^Department of Scientific Research, Shanghai Institute of Planned Parenthood Research, Shanghai, China; ^7^Dalian Institute of Chemical Physics. Chinese Academy of Sciences, Dalian, China

**Keywords:** amino acids, new predictive model, metformin, PLS, DR

## Abstract

**Objective:**

This study established a model to predict the risk of diabetic retinopathy (DR) with amino acids selected by partial least squares (PLS) method, and evaluated the effect of metformin on the effect of amino acids on DR in the model.

**Methods:**

In Jinzhou, Liaoning Province, China, we retrieved 1031 patients with type 2 diabetes (T2D) from the First Affiliated Hospital of Liaoning Medical University. After sorting the amino acids using the PLS method, the top 10 amino acids were included in the model. Multivariate logistic regression was used to analyze the relationship between different amino acids and DR. And then the effects of metformin on amino acids were explored through interaction. Finally, Spearman’s rank correlation analysis was used to analyze the correlation between different amino acids.

**Results:**

After sorting by PLS, Gly, Pro, Leu, Lyr, Glu, Phe, Tyr, His, Val and Ser were finally included in the DR risk prediction model. The predictive model after adding amino acids was statistically different from the model that only included traditional risk factors (p=0.001). Metformin had a significant effect on the relationship between DR and 7 amino acids (Gly, Glu, Phe, Tyr, His, Val, Ser, p<0.05), and the population who are not using metformin and have high levels of Glu (OR: 0.44, 95%CI: 0.27-0.71) had an additive protection effect for the occurrence of DR. And the similar results can be seen in high levels of Gly (OR: 0.46, 95%CI: 0.29-0.75), Leu (OR: 0.48, 95%CI: 0.29-0.8), His (OR: 0.46, 95%CI: 0.29-0.75), Phe (OR: 0.24, 95%CI: 0.14-0.42) and Tyr (OR: 0.41, 95%CI: 0.24 -0.68) in population who are not using metformin.

**Conclusions:**

We established a prediction model of DR by amino acids and found that the use of metformin reduced the protective effect of amino acids on DR developing, suggesting that amino acids as biomarkers for predicting DR would be affected by metformin use.

## Introduction

Type 2 diabetes (T2D) is a chronic disease characterized by elevated blood glucose levels mainly caused by insulin resistance or insufficient secretion. With the increasing development of the global economy in recent years, the prevalence of T2D has also been increasing year by year, which has become a global public health problem. According to the statistics, 537 million adults (20-79 years old) will have diabetes worldwide in 2021, which means 1 in 10 people developed diabetes (1), And this number is expected to grow by 51% by 2045 (2). Diabetic retinopathy (DR) is one of the most common complications of T2D and it is the leading cause of vision loss and preventable blindness in adults with T2D aged 20-74 years (3). In a meta-analysis of 35 studies worldwide between 1980 and 2008, the overall prevalence of DR was 34.6% (95% CI: 34.5-34.8) (4). Although the proportion of severely blinded patients has been declining among DR patients, the number of visual impairment and blindness due to DR is still on the rise because of the large increase of DR in T2D patients worldwide.

In the context of the COVID-19 epidemic, telemedicine has played an active role in the prevention and control of DR, and important innovations in diabetes treatment and monitoring have helped increase the availability of medical resources in remote areas, making it difficult for patients to seek medical care during severe epidemics; Ultimately, it can reduce the number of people blinded by DR (5).

However, the number of DR patients is still increasing, and it is still an urgent problem to be solved. At present, the research on DR has always been a hot topic. Early diagnosis of DR can effectively slow down the disease process and maximize the quality of life and survival time of T2D patients (6). Studies have found that in addition to traditional factors, metabolism and drugs can both affect the development of DR. Metformin is the first-line drug for the treatment of T2D, it can affect metabolism in the body, respent as: control blood sugar and delay/reduce the occurrence of diabetic complications (DR、DN) (7). Multiple mouse studies have found that taking metformin can improve and delay the progression of DR to a certain extent (8, 9). Although it is generally believed that metformin achieves hypoglycemic effects by inhibiting hepatic glucose output, there is now growing evidence that metformin may also act through pathways in the gut in which the drug can increase glucose consumption, stimulate GLP-1 production and alter the microbiome (10–12). Besides, population studies have found that metformin exerts diabetes treatment-related effects through AMPK and non-AMPK-mediated pathways may prevent aging and improve DR (13). An article published in nature in 2022 revealed the anti-glycemic mechanism of metformin: it believes that small doses of metformin can target the lysosomal AMPK pathway through PEN2, thereby exerting its anti-glycemic effect. The fact that knockout or depletion of PEN2 attenuates the antiglycemic effect of metformin also proves that PEN2 is one of the targets of metformin action (14).

The pathogenesis of DR is a multi-mechanism and multi-factor process. However, when analyzing correlated biomarkers with the statistical methods we usually use, many statistical results of actual important factors cannot be obtained correctly and effectively due to problems such as overfitting and instability (15). Machine learning is the exploration of the computer’s ability to learn without being explicitly programmed. It is an area of artificial intelligence that has been effectively used in healthcare in recent years (16). Machine learning algorithms have better generalization and differentiation in high-dimensional data, and can better reflect the real health status of patients, especially the occurrence and development of complications (17). Therefore, many researchers strive to develop various predictive models. In recent years, machine learning algorithms have been widely applied to many aspects of diabetes, and artificial intelligence using machine learning and deep learning has been adopted by many studies on T2D, but less research is done for DR (18).

Amino acids are important components of human health, but unbalanced amino acid levels are also closely related to metabolic disorders, insulin resistance (IR) or diabetes. Studies have shown that essential amino acids such as branched-chain amino acids (BCAAs) have been extensively investigated as a possible biomarker and even the cause of IR. Metabolic dysfunction, upregulation of the mammalian target of the rapamycin (mTOR) pathway, the gut microbiome, 3-hydroxyisobutyrate, inflammation, and the collusion of G-protein coupled receptors (GPCRs) are among the indicators and causes of metabolic disorders generating from amino acids that contribute to IR and the onset of type 2 diabetes mellitus (T2DM) and complications such as DR and DN (18). In our study, we screened out the amino acids that have the greatest impact on the risk of DR among different amino acids and established a prediction model. And we evaluated the effect of metformin on the prediction of amino acids in the model.

## Materials and methods

### Study method and population

**Figure 1:**
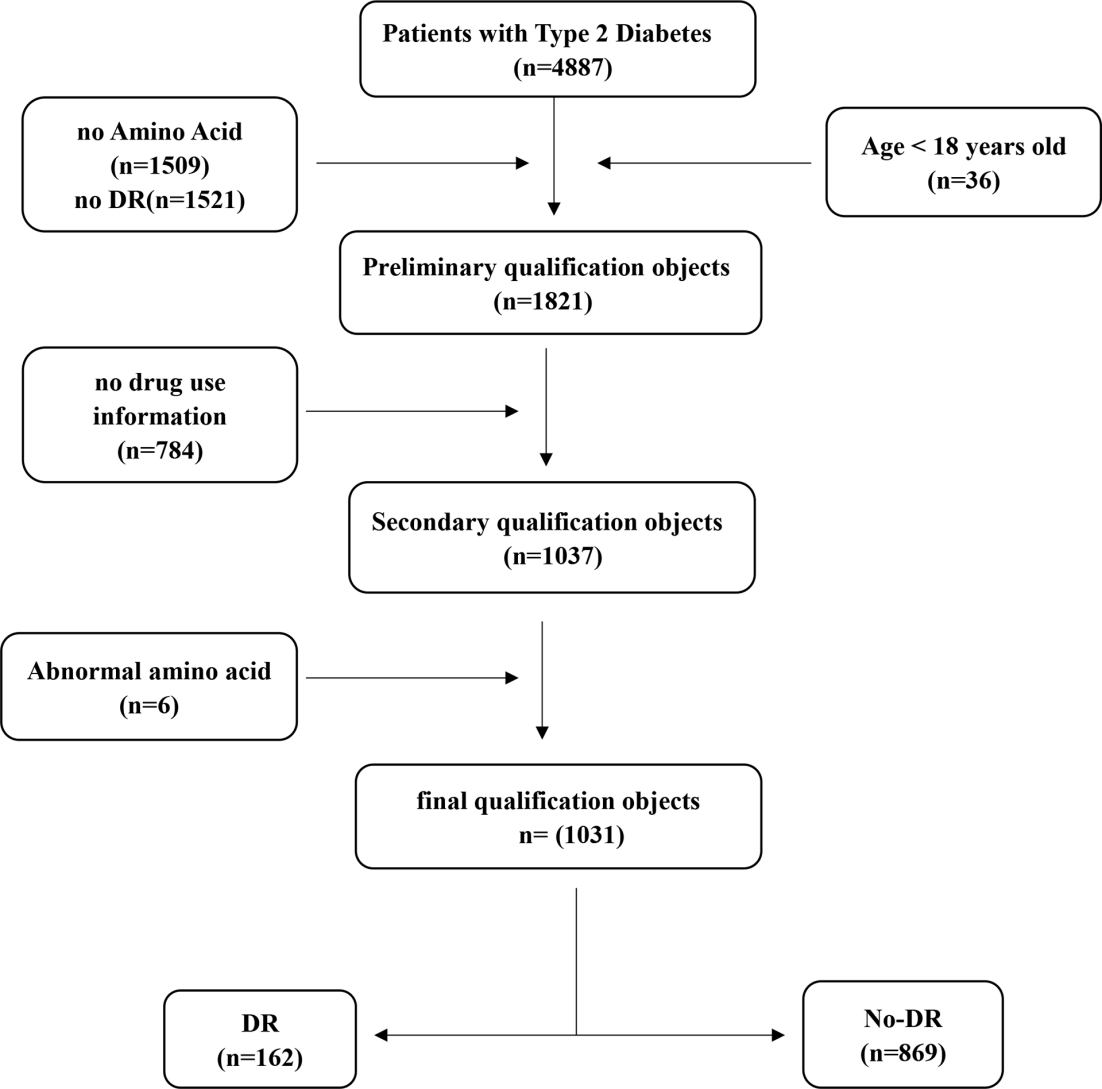
Schematic diagram of subject screening process.

The First Affiliated Hospital of Liaoning Medical University (FAHLMU) is a tertiary general hospital located in Jinzhou City, Liaoning Province, China. We performed an analysis of the electronic case here and its metabolite-related data. Inclusion criteria: 1) Patients diagnosed with T2D or treated with anti-glycemic drugs; 2) Metformin use conditions and DR prevalence information are all complete. Exclusion criteria: 1) T2D patients under the age of 18; 2) Subjects lacking the amino acid indicators, as well as height, weight, and blood pressure. A total of 1821 patients with T2D were preliminarily included in this study. According to the criteria, a total of 1031 subjects were finally included in this study, including 162 DR patients in the case group and 869 T2D patients without DR in the control group (**Figure 1**). The diagnosis and classification of T2D in the present study were based on the standard published by World Health Organization (WHO) or treated with antihyperglycemic therapy (19). Diagnostic criteria for DR based on ophthalmologic findings for T2D (20).

The Ethics Committee for Clinical Research of FAHLMU approved the ethics of the study, and informed consent was waivered due to the retrospective nature of the study, which is consistent with the Declaration of Helsinki.

### Data collection and clinical definitions

The data retrieved from the electronic medical records included demographic and anthropometric information, as well as current clinical factors and diabetic complications. Demographic included gender and age. Anthropometric measurements included height, weight, systolic blood pressure (SBP) and diastolic blood pressure (DBP). Clinical parameters included glycosylated hemoglobin (HbA1c), triglycerides (TG), total cholesterol (TC), high density lipoprotein cholesterol (HDL-C), low density lipoprotein cholesterol (LDL-C), urinary creatinine (UA), creatinine (Crea). And the duration of DR was recorded to exclude the interference of the duration of the disease on the results.

In the hospital, the measurements of anthropometric indicators were measured by using standardized procedures. Participants were allowed to wear light clothes and no shoes. Height and weight were measured to the nearest 0.5 cm and 0.1 kg, respectively. Blood pressure in adults was measured after a cuff on the right arm with a standard mercury sphygmomanometer and after a 10-minute rest in a seated position at an appropriate size. Age was obtained from the date of birth to the date of hospitalization or medical examination, and was calculated in years. The body mass index (BMI) was calculated as the ratio of body weight (kg) to squared height (m) and classified according to the overweight and obesity criteria recommended by the National Health Commission of China (21). The identification of DR is usually based on the patient’s diabetes history combined with ophthalmologic fundus examination and special examination (fundus fluorescence angiography). Microaneurysms and small hemorrhagic plaques are the earliest and relatively accurate signs of retinopathy. DR was assessed by bilateral retinal photographs and defined as present if the following lesions were found: microaneurysm, retinal hemorrhage, soft exudate, hard exudate, or vitreous hemorrhage (20).

Based on the RCS curves, we divided the total population into two groups according to different amino acids. The concentration division nodes of Glu, Gly, Leu, His, Phe and Tyr *in vivo* are 98 μmol/L, 196 μmol/L, 128 μmol/L, 51 μmol/L, 47 μmol/L and 47 μmol/L, respectively. The binary classification amino acids are then used for the following two-factor additive interaction analysis.

### Amino acid quantification

Details of the metabolomics assessment method were published previously (22). Briefly, 8 h of fasting blood sample was collected at admission. A total of 23 amino acids, i.e., alanine (Ala), asparagine (Asn), leucine (Leu), phenylalanine (Phe), tryptophan (Trp), tyrosine (Tyr), valine (Val), arginine (Arg), glycine (Gly), proline (Pro), threonine (Thr), citrulline (Cit), glutamine (Gln), histidine (His), lysine (Lys), methionine (Met), serine (Ser), ornithine (Orn), glutamate (Glu), aspartate (Asp), piperamide (Pip), cysteine (Cys), Homocysteine (Hcy) were detected *via* LC-MS. AB Sciex 4000 QTrap system (AB Sciex, Framingham, MA, USA) was used to conduct direct injection MS metabolomic analysis. Analyst v1.6.0 software (AB Sciex) was used for data collection. ChemoView 2.0.2 (AB Sciex) was used for data preprocessing. Isotope-labeled internal standard samples were purchased from Cambridge Isotope Laboratories (Tewksbury, MA, USA). Standard samples of the amino acids were purchased from Chrom Systems (Grafelfing, Germany).

### Statistical analysis

Continuous data were expressed as mean ± standard deviation (SD), non-normally distributed data were expressed as median (interquartile range), and categorical variables were expressed as numbers (percentages). It was tested whether there were differences between the different indicators of the patients in different groups. Continuous variables were normally distributed with t-test or ANOVA, non-normal variables were analyzed by nonparametric test, and categorical variables were analyzed by chi-square test.

Before building the model, the dataset is divided using random under-sampling to generate training set and test set. The test set was used to evaluate the ability of the machine learning model to predict concurrent DR in T2D patients, and feature selection was performed on the training set. We screened the amino acid ranking map of the effect on the risk of DR by partial least squares (PLS) method, and selected the top 10 amino acids for further analysis.

Binary logistic regression models were used to obtain odds ratios (OR) and the 95% confidence intervals (95%CI). Traditional risk factors for DR in T2D patients adjusted through structural adjustment programs: the model adjusted age, gender, body mass index, systolic blood pressure, diastolic blood pressure, low-density lipoprotein cholesterol, high-density lipoprotein cholesterol, triglyceride, total cholesterol, glycosylated hemoglobin, Duration of DR, uric acid and creatinine. Through the analysis, we obtained the unadjusted OR value and the adjusted OR value after adding traditional risk factors in the total population and in different populations with different drug use conditions. Restrictive cubic spline (RCS) is a curve that can provide a more intuitive relationship. According to the change of the RCS curve, we select 3 nodes in the RCS. We used it to obtain cut-off values for metabolites associated with DR risk, and selected a cut-off point by visually inspecting the curves of DR probability. Spearman correlation analysis confirmed the correlation between special amino acids, and then the additive interactions between different amino acids and metformin were then analyzed. Finally, a new model was established with the selected amino acids through the test set. Then comparing with the model that only include traditional risk factors.

All analyses were performed using R version 4.1.0 and SAS 9.4.

## Result

### Description of study subjects

**Table 1** summarizes the selection characteristics of the total population, the DR group and the control group respectively. A total of 1031 participants were included in the study, with a mean age of 57.24 years old (SD: 13.82) and a mean BMI of 25.29 (SD: 3.85). There were 548 males (53.15%) in the total population. Diabetic Nephropathy (DN) was present in 188 patients (18.2%) in the total population. In addition to 358 patients (34.7%) using metformin, 364 patients (35.3%) were using Acarbose and 146 patients (14.2%) were using Sulfonylureas.

**Table 1:** Clinical and biochemical characteristics of participants according to the occurrence of diabetic retinopathy.

Variables	Total People Mean/number (SD or %)	Non-DR Mean/number (SD or %)	DR Mean/number (SD or %)	Pα
Age(years)	57.24±13.82	57.14 ±14.43	57.77 ±9.96	0.592
Male sex	548(53.15)	475 (54.7)	73 (45.1)	0.031
Weight(kg)	70.34±13.18	70.61±13.36	68.89 ±12.09	0.126
Height(cm)	167.00(160.00, 172.00)	167.00 (160.00, 173.00)	164.00 (160.00, 172.00)	0.041
BMI(kg/m²)	25.29±3.85	25.33±3.95	25.09±3.31	0.464
SBP (mmHg)	140.39±23.99	139.42±23.63	145.60±25.26	0.003
DBP (mmHg)	82.43±13.50	82.32±13.51	83.04 ±13.44	0.532
HbA1C(%)	9.30 (7.70, 11.00)	9.30 (7.70, 11.00)	9.25 (7.70, 10.90)	0.616
Triglyceride (mmol/L)	1.69 (1.13, 2.39)	1.69 (1.12, 2.39)	1.69 (1.18, 2.40)	0.621
TC(mmol/L)	4.64(3.86, 5.29)	4.61 (3.83, 5.25)	4.81 (4.07, 5.59)	0.003
HDL-C(mmol/L)	1.02(0.85, 1.25)	1.01 (0.85, 1.25)	1.04 (0.88, 1.29)	0.141
LDL-C (mmol/L)	2.78(2.19, 3.36)	2.77 (2.15, 3.34)	2.87 (2.34, 3.45)	0.035
UA	311.00(245.95, 381.50)	310.00 (244.00, 383.00)	314.50 (251.25, 373.02)	0.602
Crea	58.97 (49.02, 73.30)	59.49 (49.09, 73.55)	56.81 (48.76, 71.37)	0.441
Diabetic Nephropathy	188 (18.2)	124 (14.3)	64 (39.5)	<0.001
Metformin	358(34.7)	307 (35.3)	51 (31.5)	0.393
Acarbose	364 (35.3)	311 (35.8)	53 (32.7)	0.508
Sulfonylureas	146 (14.2)	117 (13.5)	29 (17.9)	0.172

Pα is obtained by comparing the two groups divided by the prevalence of DR.

DR Diabetic Retinopathy, BMI, body mass index, SBP, systolic blood pressure. DBP, diastolic blood pressure; HbA1c, glycated hemoglobin; TG, triglyceride; HDL-C, high-density lipoprotein cholesterol; LDL-C, low-density lipoprotein cholesterol; UA, uric acid; Crea creatinine, DN Diabetic Nephropathy.

Data are mean ± standard deviation, median (IQR), or n (%).

P values were derived from the t-test for normally distributed variables, Mann-Whitney U test for skewed distributions, Chi-square test (or fisher test if appropriate) for categorical variables. P < 0.05 was defined as statistically significant.

After comparing the two groups, it was found that except for gender, height, SBP, TC, LDL-C and the prevalence of DN, the difference between the other indicators was not statistically significant. Among the DR patients included in this study, there were more women patients in this group, and the patients were shorter in height and higher in SBP, TC, and LDL-C. And the proportion of DN in DR patients (39.5%) was significantly higher than that of T2D patients without DR (14.3%) .

### Sorting of amino acids and the association between amino acids and DR

**Figure 2:**
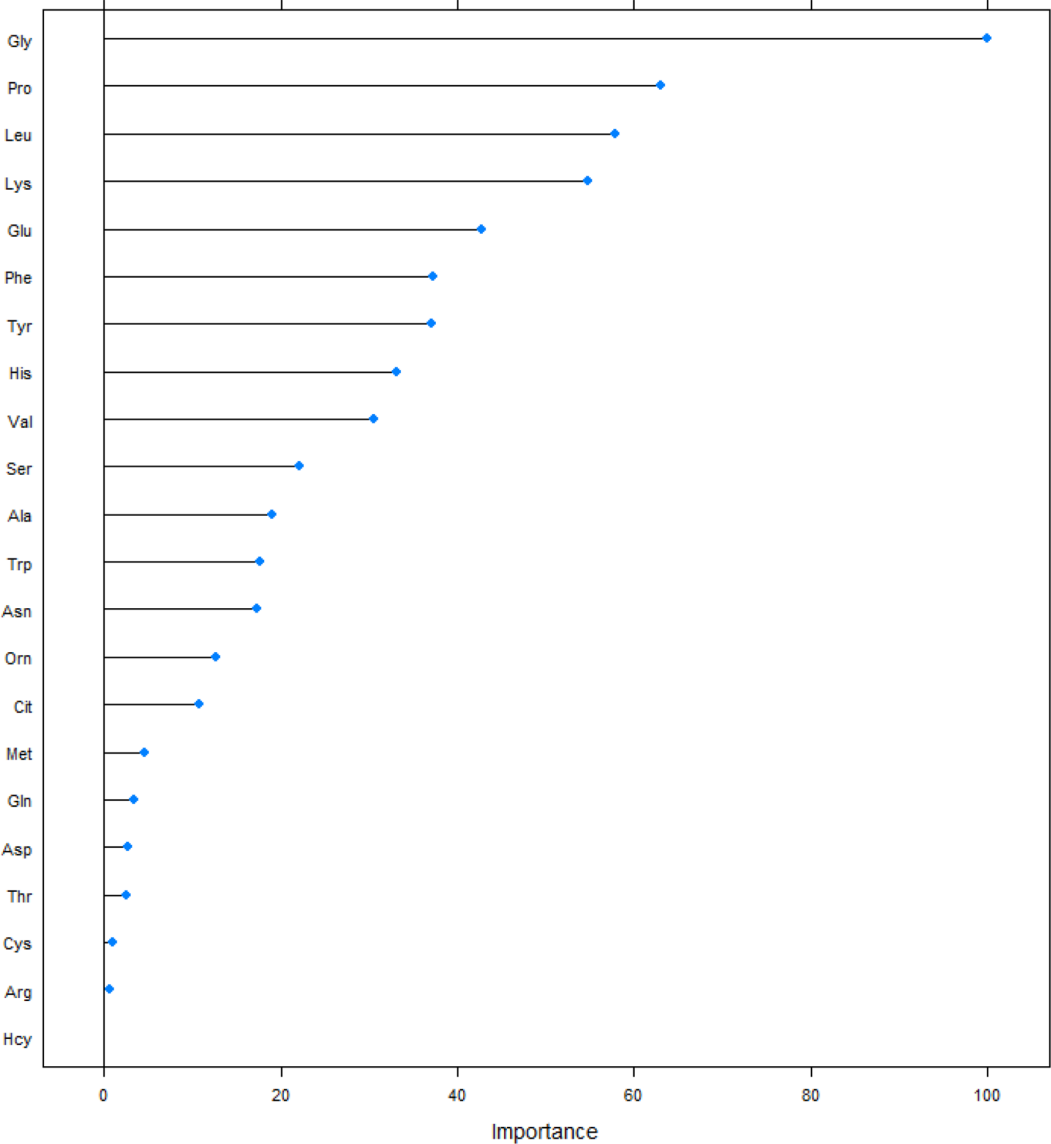
Sorting plot of amino acids of the magnitude of the effect of partial least-square method on the risk of DR. Ala, alanine; Asn, asparagine; Leu, leucine; Phe, phenylalanine; Trp, tryptophan; Tyr, tyrosine; Val, valine; Arg, arginine; Gly, glycine; Pro, proline; Thr, threonine; Cit, citrulline; Gln, glutamine; His, histidine; Lys, lysine; Met, methionine; Ser, serine; Orn, ornithine; Glu, glutamate; Asp, aspartate; Pip, piperamide, Cys, cysteine, Hcy, Homocysteine.

We screened the included amino acids using the PLS method, and obtained a ranking of the importance of amino acids on the development of DR (**Figure 2**). In included amino acids, Gly, Pro, and Leu had the greatest impact on the risk of DR, ranking in the top 3. Next are Lys, Glu, Phe, Tyr, His, Val and Ser (top 10).

According to **Figure 2**, we selected the top 10 amino acids into the model and performed logistic regression analysis on the risk of DR (**Table 2**). In the general population, the protective effect of Gly, Leu, Glu, Phe, Tyr, His, Val, Ser on the risk of DR was statistically significant, univariate analysis and multivariate analysis are all the same.

**Table 2:** Logistic regression of different amino acids and DR under the action of Metformin.

	Univariable Model	P	Multivariable Model	P
Gly, µmol/L	0.7 (0.57,0.85)	< 0.001	0.62 (0.49,0.79)	< 0.001
Pro, µmol/L	0.88 (0.74,1.05)	0.156	0.82 (0.67,1.01)	0.052
Leu, µmol/L	0.69 (0.56,0.84)	< 0.001	0.64 (0.5,0.81)	< 0.001
Lys, µmol/L	1.08 (0.93,1.26)	0.311	1.09 (0.91,1.3)	0.368
Glu, µmol/L	0.72 (0.59,0.88)	< 0.001	0.66 (0.52,0.84)	< 0.001
Phe, µmol/L	0.46 (0.36,0.59)	< 0.001	0.41 (0.31,0.56)	< 0.001
Tyr, µmol/L	0.53 (0.43,0.67)	< 0.001	0.5 (0.39,0.65)	< 0.001
His, µmol/L	0.77 (0.62,0.94)	0.007	0.73 (0.57,0.93)	0.006
Val, µmol/L	0.79 (0.66,0.95)	0.008	0.77 (0.62,0.96)	0.015
Ser, µmol/L	0.72 (0.59,0.89)	0.001	0.68 (0.52,0.87)	< 0.001

DR, Diabetic Retinopathy; Phe, phenylalanine; Tyr, tyrosine; Val, valine; Gly, glycine; Pro, proline; His, histidine; Lys, lysine; Ser, serine; Glu, glutamate, Leu Leucine.

Multivariable Model was adjusted for age, gender, body mass index, systolic blood pressure, diastolic blood pressure, triglyceride, total cholesterol, low-density lipoprotein cholesterol, high-density lipoprotein cholesterol, glycosylated hemoglobin, Duration of DR, uric acid, serum creatinine.

### Interaction between amino acids and metformin

**Table 3** shows the amino acids with statistically significant additive interactions with metformin and their addictive interaction coefficients. High levels of Glu and the absence of metformin (OR: 0.44, 95%CI: 0.27-0.71) had an additive protective effect on the development of DR compared with the general population. At high levels of Gly *in vivo*, the protective effect of Gly on DR was greater, and the additive interaction became the strongest in the absence of metformin (OR: 0.46, 95%CI: 0.29-0.75). Similarly, high concentrations of Leu (OR: 0.48, 95%CI: 0.29-0.8), His (OR: 0.46, 95%CI: 0.29-0.75), Phe (OR: 0.24, 95%CI: 0.14-0.42) and Tyr (OR: 0.41, 95%CI: 0.24-0.68) had also enhanced protection against the risk of DR in the absence of metformin compared with the general population.

**Table 3:** Two-factor additive interaction between amino acids and Metformin.

	OR (95% CI)	P value
Additive interaction model of Glu and Metformin		
Glu<98 and No-Metformin	Reference	
Glu<98 and Metformin	0.67 (0.38, 1.18)	0.159
Glu≥98 and No-Metformin	0.44 (0.27, 0.71)	< 0.001
Glu≥98 and Metformin	0.61 (0.33, 1.13)	0.108
RERI	0.431 (0.077, 0.784)	
APAB	0.262 (0.104, 0.420)	
S	2.996 (0.693, 12.962)	
**Additive interaction model of Gly and Metformin**		
Gly<196 and No-Metformin	Reference	
Gly<196 and Metformin	0.77 (0.47, 1.27)	0.299
Gly≥196 and No-Metformin	0.46 (0.29, 0.75)	0.001
Gly≥196and Metformin	0.57 (0.34, 0.98)	0.036
RERI	0.463 (0.102, 0.824)	
APAB	0.275 (0.119, 0.432)	
S	3.101 (0.695, 13.824)	
**Additive interaction model of Leu and Metformin**		
Leu<128 and No-Metformin	Reference	
Leu <128 and Metformin	0.85 (0.54, 1.34)	0.485
Leu≥128 and No-Metformin	0.48 (0.29, 0.8)	0.004
Leu≥128 and Metformin	0.71 (0.44, 1.15)	0.153
RERI	0.376 (0.0753, 0.676)	
APAB	0.263 (0.102, 0.424)	
S	8.168 (0.005, 12833.36)	
**Additive interaction model of His and Metformin**		
His<51 and No-Metformin	Reference	
His <51 and Metformin	0.88 (0.56, 1.37)	0.559
His≥51 and No-Metformin	0.46 (0.29, 0.75)	0.001
His≥51 and Metformin	0.73 (0.45, 1.17)	0.181
RERI	0.458 (0.097, 0.818)	
APAB	0.273 (0.118, 0.429)	
S	3.100 (0.707, 13.580)	
**Additive interaction model of Phe and Metformin**		
Phe<47 and No-Metformin	Reference	
Phe <47 and Metformin	0.83 (0.54, 1.3)	0.418
Phe≥47 and No-Metformin	0.24 (0.14, 0.42)	< 0.001
Phe≥47and Metformin	0.72 (0.46, 1.15)	0.166
RERI	0.427 (0.090, 0.764)	
APAB	0.275 (0.117, 0.432)	
S	4.377 (0.294, 65.066)	
**Additive interaction model of Tyr and Metformin**		
Tyr<47 and No-Metformin	Reference	
Tyr <47 and Metformin	0.81 (0.53, 1.26)	0.351
Tyr≥47 and No-Metformin	0.41 (0.24, 0.68)	< 0.001
Tyr≥47 and Metformin	0.71 (0.45, 1.12)	0.138
RERI	0.384 (0.067, 0.701)	
APAB	0.256 (0.094, 0.418)	
S	4.328 (0.213, 87.960)	

Glu Glutamate, Gly Glycine, Leu Leucine, His Histidine, Phe Phenylalanine, Tyr tyrosine, OR odds ratio, CI confidence interval, RERI risk due to interaction, AP attributable proportion due to interaction, S synergy index.

Multivariable Model was adjusted for age, gender, body mass index, systolic blood pressure, diastolic blood pressure, triglyceride, total cholesterol, low-density lipoprotein cholesterol, high-density lipoprotein cholesterol, glycosylated hemoglobin, uric acid, serum creatinine.

Significant elative excess risk due to two of interaction (RERI) > 0, attributable proportion due to interaction (AP) > 0 or synergy index (S) > 1 indicates a significant additive interact.

We also explored multiplicative interactions by stratifying the population according to whether metformin was used or not, and the results were changed compared to the general population. Among them, the protective effect of Gly, Glu, Phe, Tyr, His, Val and Ser on DR was no longer statistically significant in the population using metformin. Only the protective effect of Leu was not affected by metformin. In the population who use metformin, after stepwise adjustments to the multivariate-adjusted model, we found that it was the adjustment for Crea that made the relationship between Leu and DR significant **Table 4**.

**Table 4:** Logistic regression of different amino acids and DR under the action of Metformin.

	Metformin	P	No-Metformin	P
Univariable Model				
Gly, µmol/L	0.73 (0.52,1.02)	0.053	0.68 (0.53,0.87)	< 0.001
Leu, µmol/L	0.76 (0.54,1.06)	0.091	0.66 (0.52,0.85)	< 0.001
Glu, µmol/L	1.03 (0.77,1.39)	0.822	0.58 (0.44,0.76)	< 0.001
Phe, µmol/L	0.79 (0.57,1.08)	0.131	0.33 (0.23,0.47)	< 0.001
Tyr, µmol/L	0.72 (0.5,1.03)	0.053	0.46 (0.34,0.61)	< 0.001
His, µmol/L	0.71 (0.47,1.09)	0.077	0.78 (0.61,1)	0.034
Val, µmol/L	0.95 (0.7,1.28)	0.717	0.72 (0.58,0.91)	0.006
Ser, µmol/L	0.93 (0.69,1.27)	0.664	0.63 (0.47,0.84)	< 0.001
Multivariable Model				
Gly, µmol/L	0.81 (0.56,1.19)	0.28	0.56 (0.41,0.76)	< 0.001
Leu, µmol/L	0.63 (0.4,1)	0.036	0.64 (0.48,0.86)	0.002
Glu, µmol/L	1.06 (0.74,1.51)	0.742	0.53 (0.38,0.73)	< 0.001
Phe, µmol/L	0.8 (0.54,1.17)	0.234	0.28 (0.18,0.43)	< 0.001
Tyr, µmol/L	0.66 (0.42,1.02)	0.063	0.44 (0.32,0.61)	< 0.001
His, µmol/L	0.77 (0.49,1.2)	0.199	0.71 (0.53,0.95)	0.015
Val, µmol/L	0.9 (0.62,1.31)	0.579	0.72 (0.55,0.95)	0.014
Ser, µmol/L	1.01 (0.71,1.45)	0.936	0.54 (0.38,0.77)	< 0.001

DR, Diabetic Retinopathy, Leu, leucine; Phe, phenylalanine; Tyr, tyrosine; Val, valine; Gly, glycine; His, histidine; Ser, serine; Glu, glutamate.

Multivariable Model was adjusted for age, gender, body mass index, systolic blood pressure, diastolic blood pressure, triglyceride, total cholesterol, low-density lipoprotein cholesterol, high-density lipoprotein cholesterol, glycosylated hemoglobin, Duration of DR, uric acid, serum creatinine.

### Correlations between amino acids

**Figure 3:**
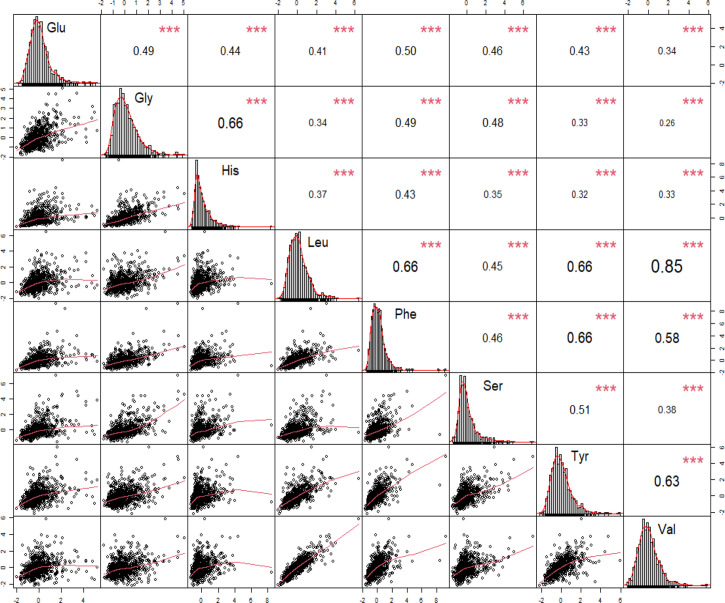
Correlations between significant amino acids for the pathogenesis of diabetic retinopathy. Leu, leucine; Phe, phenylalanine; Tyr, tyrosine; Val, valine; Gly, glycine; His, histidine; Ser, serine; Glu, glutamate. * p-value < 0.05, ** p-value < 0.01, ***p-value < 0.001.

We performed correlation analysis and fitting curve graphs on the 8 amino acids that were significantly related to DR among the selected amino acids (**Figure 3**). The results showed that all amino acids were positively correlated and the correlations were all statistically significant. Among them, the correlation between Leu and Val is the strongest, reaching a strong correlation level (r=0.85). Due to the significant correlations between amino acids, we also took the interactions into account when incorporating into the model.

### ROC model for predicting the risk of DR

**Figure 4:**
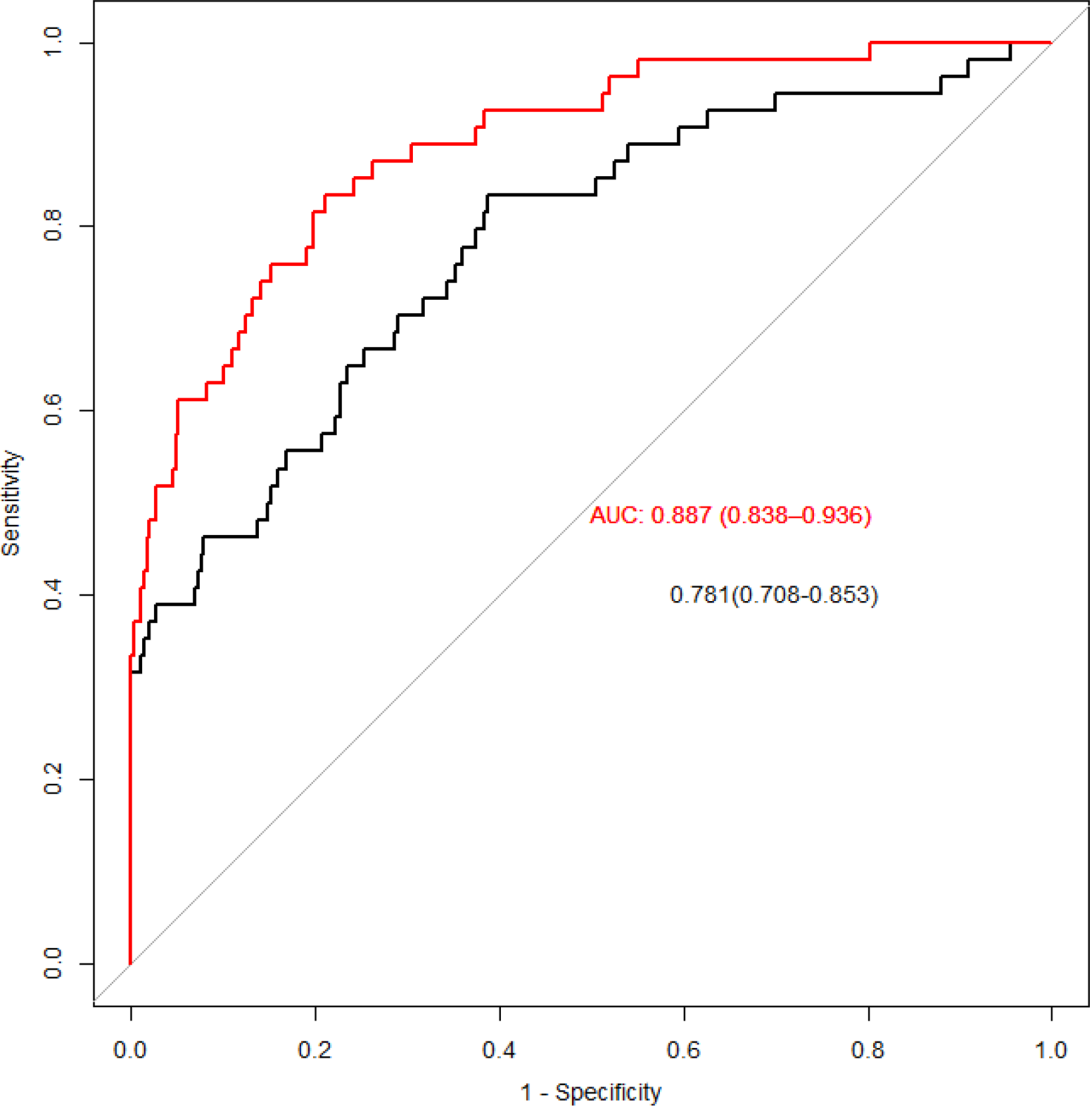
ROC curves for traditional risk factors and specific amino acids added. The red line is obtained by adding metabolites, and the black line is obtained by only traditional risk factors.

The ROC curve was fitted to the test set, showing the change in the Area Under Curve (AUC) when only traditional risk factors (black curve) and metabolites were included (red curve) (**Figure 4**). After included the amino acids we screened out by the PLS, the AUC increased from 0.781 to 0.887, and the difference between the two ROC curves was statistically significant after the Delong difference test (p=0.001). And we show the results of the model Confusion matrix in **Table 5**.

**Table 5:** Confusion matrix metrics oftest data.

	Fl_score	Precision	Accuracy	Sensitivity	Specificity
Test data(n=343)	0.9404	0.9016	0.895	0.9827	0.4259

Fl_score: Harmonic mean of Precision and Sensitivity

Precision: The ratio of the number of correctly classified positive samples to the total number of positive samples divided by the classifier. Accuracy: The proportion of correctly classified samples to all samples.

Sensitivity: The ratio of the number of correctly classified positive samples to the number of positive samples. Specificity: The ratio of the number of correctly classified negative samples to the number of negative samples.

## Discussion

At present, most of researches on the risk of DR only use the logistic regression model alone. But the logistic regression is a linear model, and the high correlation between the variables will distort the weight parameter estimation of the model (15). Due to the complexity of human metabolism and the interconversion between different amino acids, simple analysis of different amino acids may lead to collinearity problems. PLS regression is suitable when multiple independent variables have multicollinearity, finding a linear regression model by projecting predictors and observations into a new space. When establishing a model, this method will not delete the included independent variables, but will sort all the independent variables, so as to see the risk of the outcome by different independent variables. We obtained that among the amino acids, except for Pro and Lys, the effect on DR was no longer statistically significant for the top amino acids started from Ala at position 11. The roles of amino acids involved in this study on the risk of DR were mainly contributed by Gly, Pro, Leu, Lys, Glu, Phe, Tyr, His, Val and Ser, which can explain 60% of the contribution.

After the logistic analysis of the selected amino acids, it was found that Pro and Lys ranked top in the developing of DR (**Figure 2**). And although the inclusion of the two amino acids in the model will also increase the predictive ability of the model, the direct effect on the risk of DR is not statistically significant. We believe that this phenomenon is the result of the combined effect of amino acids on the pathogenesis of DR. Pro is formed by Glu enzymatically in the human body. Lys in mitochondria combines with ketoglutarate under the catalysis of LKR activity to form yeast amino acid, which is then decomposed into Glu under the catalysis of SDH activity. The metabolism of Pro and Lys is affected by Glu and enzymes. Glu is associated with T2D, and studies have shown a 0.25 drop in Glu levels in diabetic subjects compared to non-diabetic controls (23). At the same time, studies have found that Glu is a risk factor for the incidence of DR (24), while another Korean study found that the intake of Glu did not affect the incidence of DR (25). We believe that in addition to the heterogeneity of the population, the metabolic interactions between amino acids should also be taken into account. The differences of enzymes and the influence of amino acid metabolic change mask the direct role of Pro and Lys, but their predictive role as a biomarker for DR development cannot be ignored.

Our predictive model is designed to help doctors understand intestinal metabolism reflected by different amino acids on the risk of DR in diabetic patients, so as to conduct more targeted health management for different patients. Diversity of amino acid metabolism in the complex environment of the microbiota-rich gut can have beneficial or detrimental effects on the host (26). Gut microbiota can participate in the metabolism of amino acids and play a crucial role in promoting the regulation of amino acid digestion and absorption, which can affect human health (27). Resident bacterial species in the gut can also influence free amino acid distribution in the gastrointestinal tract (28). In addition, multiple studies have pointed to a causal relationship between gut microbiome dysbiosis and T2D, found that there is dysregulation of the gut microbiota in DR patients with diabetes,and DR patients exhibited enrichment of gut microbiota components at the genus level (29–31).After studying of T2D complications, it is found that the significant differences in gut microbiota composition not only between the DR and healthy groups, but also between the DR and T2D groups (29). Gut microbiota, not only worked in amino acids metabolism, but also in several other process, one of the most important is the modulation of the sensitivity to peripheral insulin. According to the bacterial flora, we may assist to an improvement of insulin resistance, with an amelioration of diabetic complications and glycemic control (32).

In addition, we evaluated the effect of the first-line drug metformin on intestinal metabolism in our predictive model. Metformin is a multi-faceted drug with multiple sites of action and molecular mechanisms, and its antihyperglycemic effect is primarily through direct or indirect reduction of hepatic glucose production and, according to several studies, by increasing insulin sensitivity in peripheral tissues of. Furthermore, the gut has been recognized as a target organ of metformin, where the drug increases glucose consumption, stimulates GLP-1 production and alters the microbiome. Our study found that a variety of amino acids have a certain protective effect on the risk of DR in T2D patients, but the protective effect of some amino acids disappeared in the people who took metformin. It maybe because that the role of metformin on human metabolism is complex, and in the gut pathway, metformin may play the opposite role. A study points out that effects of drugs on microbes in chronic diseases may confound conclusions, impacting on the protection of microbes (33). And studies have found that metformin has a rapid impact on the composition and function of the gut microbiota. Newly diagnosed T2D patients treated with metformin for 2 and 4 months had significantly changed on the composition of the gut microbiota and it was suggested that the interaction between the two occurs through regulation of metal homeostasis (34). Metformin has beneficial effects on many age-related diseases, including obesity, metabolic syndrome, cardiovascular disease, cancer, cognitive decline, and mortality (12, 35). A review article reviewed 260 relevant articles, of which 53 met the inclusion criteria and were ultimately used for data analysis; the results showed that all-cause mortality was significantly lower in diabetic patients taking metformin than in non-diabetic patients (36); there also exists study suggest that metformin also has health benefits for non-diabetic patients (12).

Several studies have demonstrated the complex relationship between gut microbiota and amino acids. A study suggests that the fusobacterium and selemonas ruminantium may play an important role in amino acid metabolism in the large intestine (37). Fusobacterium (basic bacteria for Lys or Pro utilization) in the large intestine are key drivers of amino acid fermentation, while peptostreptococcus are key drivers of Glu utilization (27). Compared with gram-positive bacteria, the ratio of Lys and His in selemonas ruminantium cells were higher, and the ratio of Pro was higher in clostridium cells (37). In addition to utilizing amino acids, the gut microbiota also plays a key role in the production of amino acids. Several amino acids produced by microbial protein fermentation in the large intestine function as precursors for the synthesis of short-chain fatty acids (38, 39). Many amino acids used by anaerobic bacteria have the potential to be metabolized to acetate, including Gly, Thr, Glu, and Orn (40), while Thr, Lys, and Glu are available for butyrate synthesis. The relationship between amino acids and intestinal flora is gradually becoming a research hotspot. Due to the correlated nature of gut metabolism with multiple diseases, this relationship points to the feasibility of amino acids as biomarkers for disease prediction. In the future research work, we will continue to carry out this work to strengthen the demonstration of our research results.

Since the outbreak of COVID-19 at the end of 2019, people’s daily lives have been severely affected, and the loss of people who need to go to the hospital for medical treatment may be more severe, especially those with chronic diseases such as diabetes and high blood pressure that require long-term care. On the one hand, the high contagiousness of the new crown epidemic has made it more difficult to overcrowd hospitals in the past, and it is more difficult for patients with chronic diseases such as diabetes to seek medical treatment, which often leads to delays in illness and induces complications such as DR and DN; on the other hand, once contracted COVID19, Patients tend to experience more severe and even life-threatening symptoms. As a result, the advantages of telemedicine are highlighted, and people’s acceptance is getting higher and higher. Telemedicine has played an active role in the prevention and treatment of diabetes and its complications and is an innovation in recent years. Especially in rural and remote areas where medical treatment is inconvenient, the screening and follow-up of DR and DN patients by experts through the Internet can alleviate the shortage of medical resources to a certain extent and improve the problem of medical inequality, which is worthy of popularization and application (5, 41).

Our research has important implications for clinical practice (1). We selected specific amino acids through the PLS method to establish a new predictive model for of DR, suggesting that T2D patients should manage amino acids that have greater effects on the developing DR in a more targeted manner, so as to achieve better management effect (2). Unlike the previous report, we pointed out that metformin can also change intestinal metabolism, which may reduce the protective effect of some amino acids while improving DR. This allows us to take a more cautious view of metformin’s effect on the disease when assessing its effect on T2D and its complications. Our research also has some shortcomings. (1) First, due to the nature of the cross-sectional study, we could not prove the existence of a causal relationship between amino acid metabolism alterations and the occurrence of DR, which requires more prospective cohort studies to confirm. (2) Our case did not distinguish the types of DR. We adjusted the duration of DR to minimize the impact. (3) This was a single-center study, the type and size of the hospital may affect the results.

In conclusion, we established a predictive model for DR and found that the use of metformin reduced the protective effect of amino acids on the developing of DR, suggesting that amino acids as biomarkers for predicting DR would be affected by the use of metformin. The specific effects of metformin on the gut microbiota require further research to determine.

## Data availability statement

The raw data supporting the conclusions of this article will be made available by the authors, without undue reservation.

## Ethics statement

The studies involving human participants were reviewed and approved by The Ethics Committee for Clinical Research of FAHLMU. Written informed consent for participation was not required for this study in accordance with the national legislation and the institutional requirements.

## Author contributions

RJ and YC conceived the project, designed experiments. ZS and WL wrote the manuscript. ZS and BH analyzed data. ZS, WL and BH collected the information and contributed to the writing of this manuscript. All authors edited the final version of the manuscript. All authors contributed to the article and approved the submitted version.

## Funding

This work was supported by the Development and clinical application of mass spectrometry methods for small molecule metabolic pathways (Project No. 2021RT09), Dalian Innovation Center of Laboratory Medicine Mass Spectrometry Technology. The funders had no role in study design, data collection and analysis, decision to publish, or preparation of the manuscript.

## Acknowledgments

The authors thank all doctors, nurses and research staff at the First Affiliated Hospital of Liaoning Medical University for their participation in this study.

## Conflict of interest

The authors declare that the research was conducted in the absence of any commercial or financial relationships that could be construed as a potential conflict of interest.

## Publisher’s note

All claims expressed in this article are solely those of the authors and do not necessarily represent those of their affiliated organizations, or those of the publisher, the editors and the reviewers. Any product that may be evaluated in this article, or claim that may be made by its manufacturer, is not guaranteed or endorsed by the publisher.
